# The antidepressant-like effects of pioglitazone in a chronic mild stress mouse model are associated with PPARγ-mediated alteration of microglial activation phenotypes

**DOI:** 10.1186/s12974-016-0728-y

**Published:** 2016-10-04

**Authors:** Qiuying Zhao, Xiaohui Wu, Shuo Yan, Xiaofang Xie, Yonghua Fan, Jinqiang Zhang, Cheng Peng, Zili You

**Affiliations:** 1 School of Life Science and Technology, Center for Informational Biology, University of Electronic Science and Technology of China, Chengdu, 610054 China; 2 State Key Laboratory Breeding Base of Systematic Research, Development and Utilization of Chinese Medicine Resources, Pharmacy College, Chengdu University of Traditional Chinese Medicine, Chengdu, 6111376 China

**Keywords:** Pioglitazone, PPARγ, Antidepressant, CMS, Microglia, Alternative activation, Cytokine

## Abstract

**Background:**

Discoveries that microglia-mediated neuroinflammation is involved in the pathological process of depression provided a new strategy for novel antidepressant therapy. Peroxisome proliferator-activated receptor γ (PPARγ) is a nuclear receptor regulating inflammation and microglial polarization and, therefore, a potential target for resolving depressive disorders. Our hypothesis was that antidepressant effects could be achieved through anti-inflammatory and neuroprotective activities by PPARγ-dependent microglia-modulating agents.

**Methods:**

Chronic mild stress (CMS) treatment was performed on C57BL/6 mice for 6 weeks. After 3 weeks with the CMS procedure, depressive-like behaviors were evaluated by sucrose preference (SP), tail suspension test (TST), forced swimming test (FST), and locomotor activity. Pioglitazone was administered intragastrically once per day for 3 weeks at different doses. Neuroinflammatory cytokines were determined by real time-PCR (RT-PCR), enzyme-linked immunosorbent assay (ELISA), and western blot. The activated microglial state was confirmed by immunohistochemistry. N9 microglial cells were subjected to lipopolysaccharide, pioglitazone, and GW9662 to discuss the phenotype of activated microglia by RT-PCR, ELISA, and western blot.

**Results:**

It was demonstrated that the PPARγ agonist pioglitazone (2.5 mg/kg) ameliorated depression-like behaviors in CMS-treated mice, as indicated by body weight (BW), the SP test, the FST, and the TST. The amelioration of the depression was blocked by the PPARγ antagonist GW9662. The expression of M1 markers (IL-1β, IL-6, TNFα, iNOS, and CCL2) increased, and the gene expression of M2 markers (Ym1, Arg1, IL-4, IL-10, and TGFβ) decreased in the hippocampus of the stress-treated mice. Pioglitazone significantly inhibited the increased numbers and morphological alterations of microglia in the hippocampus, reduced the elevated expression of microglial M1 markers, and increased the downgraded expression of microglial M2 markers in C57BL/6 mice exposed to CMS. In an in vitro experiment, pioglitazone reversed the imbalance of M1 and M2 inflammatory cytokines, which is correlated with the inhibition of nuclear factor kB activation and is expressed in LPS-stimulated N9 microglial cells.

**Conclusions:**

We showed that pioglitazone administration induce the neuroprotective phenotype of microglia and ameliorate depression-like behaviors in CMS-treated C57BL/6 mice. These data suggested that the microglia-modulating agent pioglitazone present a beneficial choice for depression.

**Electronic supplementary material:**

The online version of this article (doi:10.1186/s12974-016-0728-y) contains supplementary material, which is available to authorized users.

## Background

Major depressive disorder (MDD) is one of the most frequently occurring mental disorders and has a considerable rate of mortality [1]. The medications currently available to treat depression, including monoamine oxidase inhibitors (MAOIs), selective serotonin reuptake inhibitors (SSRIs), and tricyclic antidepressants, are predicated on the monoamine hypothesis. Clinical evidence shows that these medications produce adequate remission of depressive symptoms in only two thirds of patients [2]. Moreover, monoamine antidepressants such as SSRIs or MAOIs often have a slow onset of action and serious side effects [3]. Increasing evidence indicates that depression is accompanied by inflammatory responses [4, 5]. A recent study reported that the imbalance of pro- and anti-inflammatory cytokines plays a critical role in chronic mild stress (CMS)-induced depression [6]. Research into the role of neuroinflammatory responses has brought new insights into the potential etiologies of MDD and provided new strategies for novel antidepressant therapies.

Microglia are resident innate immune cells within the central nervous system (CNS) and play a central role in the neuroinflammatory response. Microglia populate the CNS during early fetal life and are present in a resting state in the healthy brain but readily become activated in response to brain trauma, injury, and infection [7, 8]. In clinical research, microglial activation has also been observed in patients with depression who have committed suicide [9]. Microglial activation is often categorized into classical (M1) and alternative (M2). M1 microglia may contribute to dysfunction of the neurotrophic system by expressing pro-inflammatory cytokines, such as tumor necrosis factor-α (TNFα), IL-1β, IL-6, iNOS, and CCL2 [10]. In the M1 phenotype of microglia, the activation of nuclear factor kB (NF-kB) may play a critical role in the production of proinflammatory cytokines, leading to neurotoxic outcomes [11]. The M2 phenotype, sometimes called the neuroprotective microglial phenotype, releases different mediators including Ym1, arignase1 (Arg1), IL-4, IL-10, and TGFβ [10] to antagonize inflammation-induced damage in the CNS [12, 13]. Stress triggers neuroinflammatory processes in several brain regions including the frontal cortex, hypothalamus, and hippocampus [14, 15]. More specifically, the hippocampus is an area particularly sensitive to chronic stress, which was associated with evidence of inflammation characterized by microglial activation [16]. In this study, the hippocampus was chosen for investigation because of the effects of hippocampal microglial activation on stress-induced behaviors.

Pioglitazone is a highly selective agonist for PPARγ, which causes the transcription of several genes involved in glucose and lipid metabolism as well as with the production of inflammatory mediators [17, 18]. In recent years, researchers have found that PPARγ agonists might be useful in a number of central nervous system diseases that have a microglia-induced anti-inflammatory response [19, 20]. Pioglitazone is primarily used as an antidiabetic drug [21] and recently has been used in clinical trials for psychiatric and neurodegenerative diseases. It has been reported that pioglitazone promotes cognitive and functional improvements in schizophrenia and autism [22]. Pioglitazone is also being tested in Alzheimer’s disease [23], Parkinson’s disease [24], multiple sclerosis [25], and stroke [26]. In clinical trials, pioglitazone worked as an adjuvant treatment for depression in insulin-resistant subjects and was associated with improvement in glucose metabolism [27]. Researchers have also reported that pioglitazone had antidepressant-like effects in an acute animal model of depression, although the mechanisms underlying their beneficial effect remain largely unknown [28, 29]. Accumulating evidence indicates that inhibition of the microglia-mediated inflammatory response is an important strategy for the treatment of mood disease [30].

Based on the effects of PPARγ on the anti-inflammatory response, in this study we evaluated the antidepressant-like effects of the PPARγ agonist pioglitazone in CMS-induced depression using a mouse model and analyzed the role of M2 microglia in the antidepressant activity of pioglitazone.

## Methods

### Animals

Male C57BL/6 mice weighing 18–22 g were purchased from the Institute of Experimental Animals (Chengdu, China). The animals were housed individually under controlled conditions (23–25 °C, humidity 50–60 %, on a 12-h light/dark cycle with lights on at 8:00 a.m. and water and food available ad libitum) and acclimatized for at least 1 week before they were used for the experiments. All the behavioral experiments were conducted in isolated behavioral testing rooms and performed by an experimenter who was blind to the identity of the experimental groups.

### CMS procedure

The CMS procedure was performed according to previously described methods, but with small modifications [31, 32]. The stress method followed a random schedule of commonly used mild stressors, such as food deprivation for 24 h, water deprivation for 24 h, cage tilting (45°) for 24 h, damp bedding for 24 h, stroboscopic illumination for 24 h, overnight illumination, and restraint stress for 2 h. The mice received one stressor once per day with the same stressor never applied in two consecutive days. The stress regimen lasted for six consecutive weeks. The control group was housed in a separate room and had no contact with the stressed animals. After the 3 weeks with the CMS procedure, the stress-treated mice were divided into a vehicle group and drug-treatment groups. The body weight (BW) and sucrose preference (SP) data during the first 3 weeks while the mice were being stressed showed no statistical differences between the mice. The additional files showed this in more details (see Additional file 1 and Additional file 2).

### Drug treatment

Depressive-like behaviors appeared after the 3 weeks of CMS induction. At the end of the 3-week induction period, the drugs and vehicle were administered intragastrically once per day for an additional 3 weeks. In order to determine the optimal dosage, for experiment 1, the mice were allocated into six experimental groups according to treatment: Control (control group not submitted to CMS, *n =* 26), CMS + Vehicle (CMS group without pioglitazone, *n* = 26), CMS + 2.5 mg/kg (CMS animals and administered with pioglitazone at 2.5 mg/kg, *n =* 26), CMS + 5 mg/kg (CMS animals and administered with pioglitazone at 5 mg/kg, *n =* 26), CMS + 10 mg/kg (CMS animals and administered with pioglitazone at 10 mg/kg, *n =* 26), CMS + 20 mg/kg (CMS animals and administered with pioglitazone at 20 mg/kg, *n =* 26). In experiment 1, BW and SP test were evaluated in the same group (*n =* 10). The tail suspension test (TST) and locomotor activity were assessed in the same animals (*n =* 8). The forced swimming test (FST) was estimated in the six groups (*n =* 8). In all the experiments, the vehicle was 10 % dimethyl sulfoxide diluted with 0.9 % saline.

In order to investigate the possible involvement of a PPARγ pathway, for experiment 2, the mice were allocated into five experimental groups according to treatment: control (control group not submitted to CMS, *n =* 26), CMS + Vehicle (CMS group and 0.9 % saline, *n =* 26), CMS + Piog (CMS animals and administered with pioglitazone at 2.5 mg/kg, *n =* 26), CMS + GW (CMS animals and administered with GW, the mice were pretreated for 1 h with 1 mg/kg of GW, a specific PPARγ inhibitor, before pioglitazone administration, *n =* 26), CMS + Piog + GW (CMS animals and administered with pioglitazone at 2.5 mg/kg and GW at 1 mg/kg, *n =* 26). In experiment 2, BW and SP test were evaluated in the same animals (*n =* 10). The TST and locomotor activity were assessed in the same mice (*n =* 8). The FST was estimated in the five group of animals (*n =* 8). The immunofluorescence experiment was performed for microglial phenotype in the control (*n =* 5), CMS + Vehicle (*n =* 5), CMS + Piog (*n =* 5), CMS + GW (*n =* 5), and CMS + Piog + GW (*n =* 5) animals. The relative expression of the M1 and M2 markers was quantified by real time-PCR (RT-PCR) in all of the five groups (*n =* 5 for each group). The pioglitazone (Sigma, USA) and GW9662 (Sigma, USA) were all diluted with 10 % dimethyl sulfoxide (Sigma, USA) in 0.9 % saline. Thirty minutes after the last drug administration, the mice were used in the behavioral tests as well as in cellular and molecular experiments.

### Body weight measurement and sucrose preference test

As the primary assessment indicators for BW and anhedonia in an animal model of depression, BW and SP were measured at the onset of the CMS schedule and every week until the end of the 6-week CMS test. BW was measured once a week at 5:00 p.m. on Mondays to calculate the BW gain during the CMS period. The BW test and the SP test were administered to all the mice in all the groups.

Before the SP test, individually housed mice were habituated to consume 1 % sucrose solution. The test involved 20 h of food and water deprivation, followed by 1 % sucrose and water intake for 2 h. The position of the two bottles (left/right sides of the cages) was varied randomly in each trial. The intake of sucrose solution and water was recorded every week at 5:00 p.m. on Tuesdays for 6 weeks throughout the CMS experiment to evaluate the anhedonia of the mice. The SP was calculated according to the ratio: SP = sucrose intake (g)/[sucrose intake (g) + water intake (g)].

### Locomotor activity measurement

To measure the locomotor activities of the mice, we used a 36-point infrared ray passive sensor system (model No. ZZ-6, Taimeng Tech Ltd. Chengdu, China) in the sixth week of the experimental procedure, that is, at the end of the test. Each of the mice was placed in a chamber of an autonomous movement device to accommodate to the environment for 1 min before the test. The total locomotor activity (standing and movement) of the mice was automatically recorded by the autonomous movement equipment for 10 min.

### Tail suspension test

The TST is based on the fact that mice subjected to the short-term, inescapable stress of being suspended by their tail will develop an immobile posture [33]. In this test, which was also done once to all mice in the sixth week of the experiment, adhesive tape (approximately 1 cm from the tip of each mouse’s tail) was used to suspend the mice by the tail from a ledge 20 cm above a tabletop. Each animal was isolated to avoid interference during the experiment. The mice were recorded by a video camera. Immobility was defined as the absence of movement for 6 min and was used as an evidence of hopelessness.

### Forced swimming test

The FST models depressant-like behavior as decreased immobility. We used the method described by Porsolt [34] with a slight modification. The mice were placed in an open glass cylindrical container (20 cm in height and 14 cm in diameter) with 10-cm-deep water (25 ± 1 °C) for 6 min. Immobility time was recorded as the amount of time the mice floated passively without struggling in the water. This test was also done once to all mice in the sixth week of the experiment. The duration of immobility in the last 4 min of the total 6 min was recorded as evidence of despair.

### Immunofluorescence

The animals were anesthetized with pentobarbital sodium, perfused transcardially with pH 7.2 phosphate-buffered saline (PBS) and 4 % paraformaldehyde. Coronal sections were cut into 35-μm slices with a sliding vibratome (CM1900; Leica Microsystems, Wetzlar, Germany). The immunofluorescence and statistical methods were based on our previously described methods [35]. The brain sections that contained the hippocampus and N9 microglial cells were permeabilized with 0.5 % Triton X-100 for 10 min. The samples were placed in 10 % donkey serum for 2 h; incubated with a primary antibody, goat anti-ionized calcium-binding adaptor protein-1 (Iba1) (1:600; Abcam), overnight at 4 °C; and then incubated with a fluorescent-dye-conjugated secondary antibody, DyLight 549-conjugate donkey anti-goat (1:500; Jackson ImmunoResearch). Positive cells were manually counted under a ×40 objective microscope (Olympus BX51). The photomicrographs were saved as TIF files and quantitatively analyzed using the cell counter in the ImageJ software (version 1.45J; National Institutes of Health, Bethesda, MD, USA).

### Cell culture

Murine N9 microglial cells (kindly provided by Dr. H. Han, Institute of Neuroscience, Fourth Military Medical University, China) were cultured in DMEM (Invitrogen, USA) with the addition of 10 % fetal bovine serum (Invitrogen, USA), 100 U/ml penicillin (Invitrogen, USA), and 0.1 mg/ml streptomycin (Invitrogen, USA) in a humidified atmosphere of 5 % CO_2_ and 95 % air at 37 °C. The cells were passaged two to three times per week and then distributed into 24-well plates at a density of 5 × 10^5^ cells per well. The cultivated N9 cells were treated with or without lipopolysaccharide (LPS) (for simulating an organism’s inflammatory environment in vitro), pioglitazone, and GW9662. The concentrations were as follows: LPS (Sigma, USA): 100 ng/ml; pioglitazone (Sigma, USA): 10 μmol, and GW9662 (Sigma, USA): 1 μmol. After culturing for 24 h, the cells were used in RT-PCR, ELISA, and western blot detection.

### ELISA

The N9 cells were treated with RIPA Lysis Buffer (Beyotime Institute of Biotechnology, China) and centrifuged at 12,000 rpm for 5 min. The supernatants were collected to detect the protein levels by ELISA and western blot. The expression of IL-1β and TNFα was assayed using ELISA kits (NeoBioscience Technology Co., Ltd., China) according to the manufacturer’s protocol. The detection limits for IL-1β and TNFα were 1 pg/ml. The absorbance at 450 nm was recorded using a microplate reader.

### Western blot analysis

The protein concentrations were determined with a BCA kit (Beyotime Institute of Biotechnology, China) following the manufacturer’s guidelines. Equal amounts of protein were separated by SDS-polyacrylamide gel electrophoresis, transferred to polyvinylidene difluoride membranes (Millipore, USA), and blocked with 5 % skim milk incubated with primary antibody either overnight at 4 °C or for 1 h at room temperature. The membranes were washed and incubated with anti-rabbit IgG horseradish peroxidase-conjugated secondary antibody (1:500; Beyotime Institute of Biotechnology, China) using ECL-Plus kits (Millipore, USA) as a detection system. Densitometry was performed to quantify the signal intensity using ImageJ software (Version 1.45 J; National Institutes of Health, Bethesda, MD, USA). The primary antibodies were rabbit anti-Ym1 (1:800; Abcam, USA), rabbit anti-Arg1 (1:800; Abcam, USA), and rabbit anti-IkBα (1:1000; Cell Signaling Technology, USA).

### Real-time PCR

The mice were sacrificed by decapitation, and the hippocampus was quickly dissected out and placed in sterile tubes. The total RNA of the hippocampus and the N9 cells were harvested using Trizol reagent (Invitrogen, USA) according to the manufacturer’s instructions. The first strand complementary DNA (cDNA) was synthesized with a cDNA Synthesis Kit (TAKARA, Japan). The PCR amplifications were conducted in a 10-μl reaction volume. RT-PCR was carried out in a Bio-Rad CFX 96 at 95 °C for 10 min followed by 38 cycles of 95 °C for 3 s, 60 °C for 30 s, and 72 °C for 5 s. The relative gene expression was calculated using the Ct method, as in our previous study [12]; the loading control was β-actin. Primer sequences were as follows: IL-1β, 5′-CCAGCAGGTTATCATCATCATCC-3′, 5′-CTCGCAGCAGCACATCAAC-3’, [GenBank: NM_008361.4]; IL-6, 5′-AGAGATACAAAGAAATGATGGA-3′, 5′-AGCTATGGTACTCCACAAGACCA-3′, [GenBank: NM_031168.2]; TNFα, 5′-CAGCCGATGGGTTGTACCTT-3′, 5′-TGTGGGTGAGGAGCACGTAGT-3′, [GenBank: NM_013693.3]; iNOS, 5′-GCAGAGATTGGAGGCCTTGTG-3′, 5′-GGGTTGTTGCTGAACTTCCAGTC-3′, [GenBank: NM_010927.4]; CCL-2, 5′-CTGATGCAGGTCCCTATGGT-3′, 5′-GCAGGATTTTGAGGTCCAGA-3′, [GenBank: NM_009137.2]; Ym1, 5′-TCACTTACACACATGAGCAAGAC-3′, 5′-CGGTTCTGAGGAGTAGAGACCA-3′, [GenBank: NM_009892.3]; Arg1, 5′-GGAAGACAGCAGAGGAGGTG-3′, 5′-TATGGTTACCCTCCCGTTGA-3′, [GenBank: NM_007482.3]; IL-4, 5′-CAGCTAGTTGTCATCCTGCTCTTC-3′, 5′-GCCGATGATCTCTCTCAAGTGA-3′, [GenBank: NM_021283.2]; IL-10, 5′-GGCAGAGAACCATGGCCCAGAA-3′, 5′-AATCGATGACAGCGCCTCAGCC-3′, [GenBank: NM_010548.2]; TGFβ, 5′-GACCGCAACAACGCCATCTA-3′, 5′-GGCGTATCAGTGGGGGTCAG-3′, [GenBank: NM_011577.2]; β-actin, 5′-CCGTGAAAAGATGACCCAGATC-3′, 5′-CACAGCCTGGATGGCTACGT-3′, [GenBank: NM_007393.5].

### Statistical analysis

The data were expressed as means ± SEM. The results of the BW and SP test were analyzed with a two-way analysis of variance (ANOVA). The other statistical significances were assessed by one-way ANOVAs followed by Bonferroni’s multiple comparison test using Windows® v.17 (SPSS Inc., Chicago, USA). A value of *p* < 0.05 was considered statistically significant.

## Results

### The effects of different doses of pioglitazone on CMS-treated mice

**Fig. 1:**
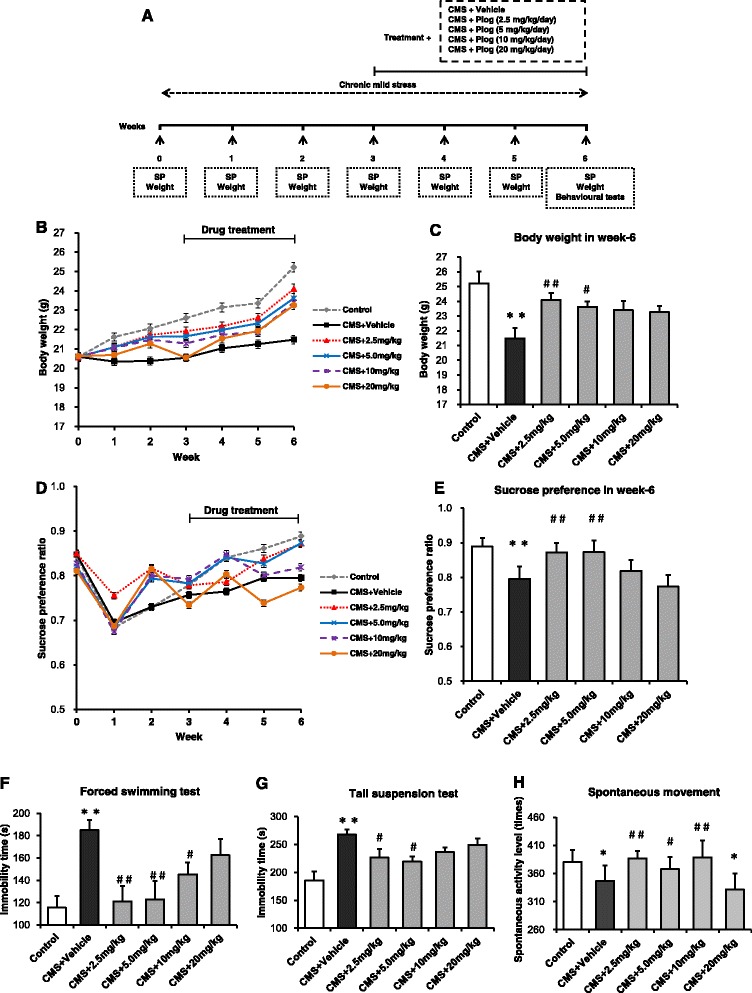
Effects of pioglitazone at different doses on CMS mice. **a** Schematic diagram of the experimental design. BW was measured every week (**b**). The 2.5 mg/kg dose showed the best result in recovering the BW in the CMS mice (**c**, *n =* 10). Anhedonia was detected by SP over the course of the 6-week-long experiment (**d**, **e**, *n =* 10). The duration of immobility in the FST (**f**) and TST (**g**) increased after CMS induction at the sixth week (*n =* 8 in both tests). Pioglitazone (2.5 mg/kg) reduced this increase. In the locomotor activity test, pioglitazone improved the spontaneous activity level after CMS induction (**h**, *n =* 8). ^*^ *p* < 0.05, ^* *^ *p* < 0.01 vs. Control; ^#^ *p* < 0.05, ^##^ *p* < 0.01 vs. CMS + Vehicle. Data are expressed as means ± SEM

The experimental design is presented in Fig. 1a. There were no differences in the BW or SP between the six groups at the baseline measurement (week 0: *p* > 0.05, respectively). The BW of the CMS mice slowly increased compared with that of the control group throughout the 3 weeks of the CMS procedure. After 3 weeks of continuous treatment with pioglitazone for the CMS mice, the doses of 2.5 and 5.0 mg/kg pioglitazone had restored the CMS-induced BW reduction in the mice to a level comparable to that of the CMS + Vehicle group (*p* < 0.01 and *p* < 0.05, respectively; Fig. 1b, c). A reduction in the relative sucrose intake (anhedonia) was observed in the mice after the 3-week period of CMS. The anhedonia improved following the 2.5 and 5.0 mg/kg dosages to a level that was comparable to that of the CMS + Vehicle mice (*p* < 0.01, both; Fig. 1d, e). Additional file 1 provides the 6-week details of BW and SP in experiment 1.

With 6 weeks of CMS induction, the immobility time increased in the CMS + Vehicle mice. Three weeks of treatment with pioglitazone at 2.5, 5.0, and 10 mg/kg significantly reduced the duration of immobility compared to the untreated CMS group (*p* < 0.01; Fig. 1f).

In the TST, after 6 weeks of CMS induction, the duration of the immobility increased in the CMS group compared with the control group. The immobility time of the pioglitazone (2.5 and 5.0 mg/kg)-treated mice was shorter than that of the CMS + Vehicle mice (*p* < 0.05; Fig. 1g).

As shown in Fig. 1h, the CMS + Vehicle group showed decreased spontaneous movement times compared with the control animals (*p* < 0.05; Fig. 1h). The spontaneous activity levels increased in the 2.5, 5.0, and 10 mg/kg pioglitazone groups compared with the stressed, but untreated, mice. According to the depression indicators above, the antidepressant effect was greatest at the 2.5 mg/kg pioglitazone level.

### The effects of pioglitazone on behaviors of CMS-treated mice blocked by GW9662

**Fig. 2:**
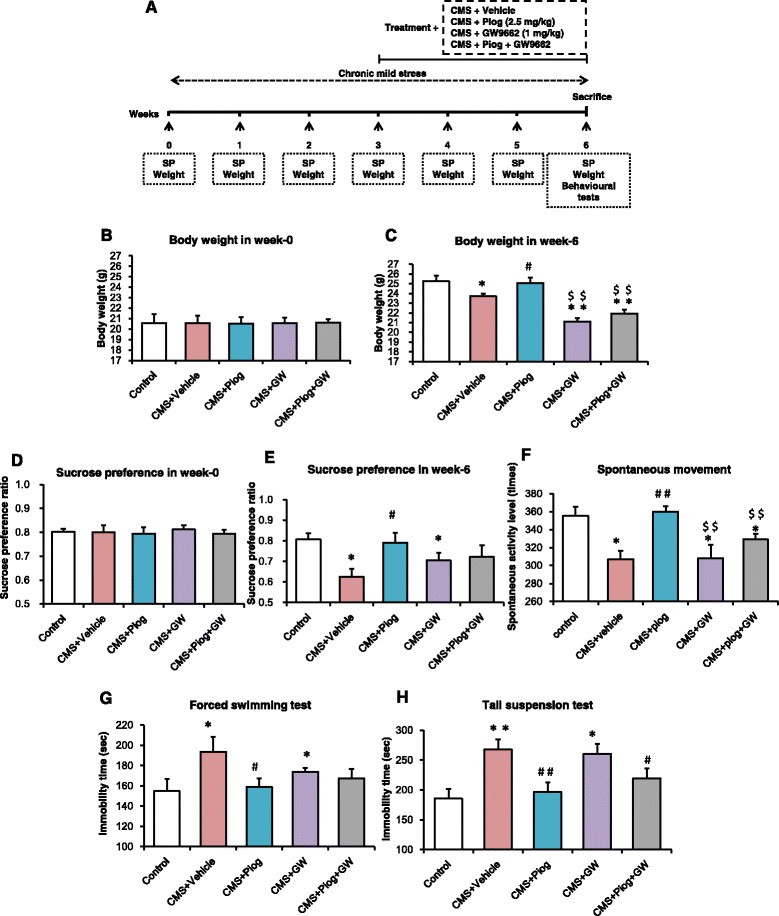
The activity of PPARγ affected the improvement of depression-like behaviors. **a** The principle scheme of the experimental project. BW at the baseline level (**b**) and the sixth week (**c**) (*n =* 10). SP showed no differences at week 0 (**d**). Pioglitazone improved the ratio of the SP in CMS mice but anhedonia was maintained with the antagonist of PPARγ, GW9662, administration (**e**, *n =* 10). Spontaneous activity was modulated by pioglitazone and GW9662 (**f**, *n =* 8). Pioglitazone decreased the time of immobility, but GW9662 increased the immobility duration in the FST and TST (**g**, **h**, *n =* 8 in both tests). ^*^ *p* < 0.05, ^**^ *p* < 0.01 vs. Control; ^#^ *p* < 0.05, ^##^ *p* < 0.01 vs. CMS + Vehicle; ^$$^ *p* < 0.01 vs. CMS + Piog. Data are expressed as means ± SEM

As shown in Fig. 2a, GW9662 was chosen to inhibit the PPARγ activity. The BW and SP baselines did not differ between the five groups (*p* > 0.05, both; Fig. 2b, d), but the CMS induction reduced the BW and SP in week 6 compared with the control group. After 3 weeks of drug administration, pioglitazone reversed the body loss and increased the sucrose intake compared with the CMS + Vehicle mice. GW9662 aggravated the weight loss and anhedonia after the CMS procedure (*p* < 0.01, *p* < 0.05; Fig. 2c, e). Mice treated with pioglitazone and GW9662 in the CMS groups also decreased in BW, but not SP, compared to the CMS + Vehicle group. Only the week 6 data for the BW and SP tests are shown in Fig. 2. Additional file 2 shows more details for the BW and SP.

The mice exposed to CMS induction showed reduced locomotor movement during the 6 weeks of stress treatment compared with the control animals. Pioglitazone increased the durations of their spontaneous movement after 3 weeks of administration, whereas GW9662 treatment decreased the spontaneous activity level compared with the controls and the CMS + Piog (*p* < 0.05). Pioglitazone and GW9662 combined did not significantly change the effect of the CMS (Fig. 2f).

In the FST, the duration of the immobility increased in the CMS + Vehicle animals, whereas the time was restored to the control level with a 3-week pioglitazone treatment. After administration with the PPARγ antagonist GW9662, the immobility time increased, compared to the control mice (*p* < 0.05; Fig. 2g). There were no significant differences in the FST between the treatment of the CMS-exposed mice with the two-drug (pioglitazone and GW9662) combination and the control group.

Figure 2h depicts the effect of PPARγ on depression improvement in the TST experiment. The immobility time of the CMS + Vehicle as well as of the CMS + GW9662 mice was longer than that of the control group. Mice that received pioglitazone either alone or in combination with GW9662 had a shorter immobility duration than the CMS + Vehicle mice (*p* < 0.05; Fig. 2h).

### The activity of PPARγ affects the microglial activated status

**Fig. 3:**
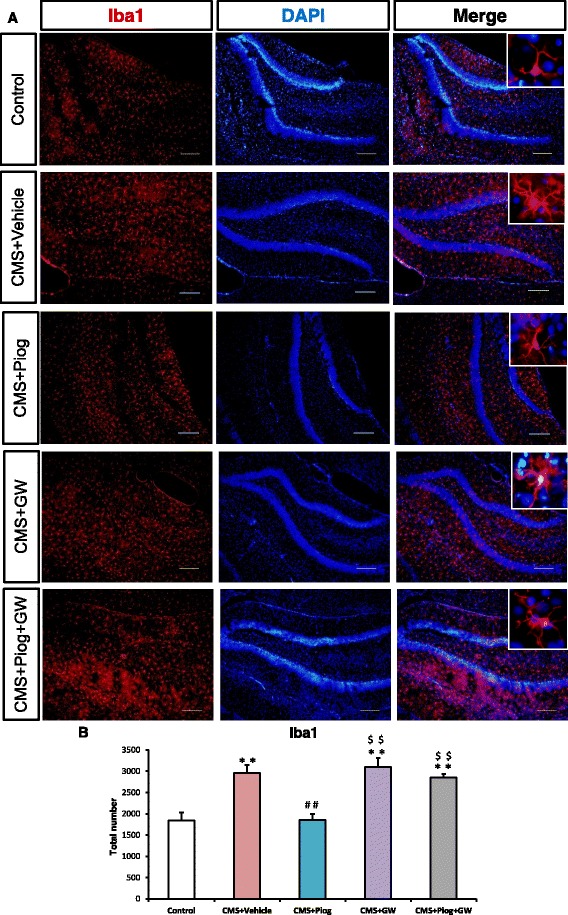
Microglial activated status was influenced by PPARγ in the hippocampus. Pioglitazone decreased the number of Iba1^+^ microglia compared with the CMS mice. The Iba1^+^ microglial morphology was amoeboid, and the number of microglia increased in the animals after GW9662 treatment. Administration of pioglitazone and GW9662 had no effect on the microglia (**b**, *n =* 5). *Enlarged figures* indicate typical microglia (**a**). *Scale bars*: 10 μm. ^**^ *p* < 0.01 vs. Control; ^##^ *p* < 0.01 vs. CMS + Vehicle; ^$$^ *p* < 0.01 vs. CMS + Piog. Data are expressed as means ± SEM

After 6 weeks of CMS induction, the morphology of the Iba1^+^ microglia was amoeboid (Fig. 3a) and their numbers increased in the hippocampus of the CMS + Vehicle mice compared with the control animals (Fig. 3b). The number of Iba1^+^ microglia in the mice which received the CMS induction was lower in the animals that received the PPARγ agonist, pioglitazone. The number of Iba1^+^ microglia increased and their amoeboid shape remained following the administration of the PPARγ antagonist GW9662. Pioglitazone and GW9662 treatment together did not change the number or morphology of the Iba1^+^ microglia compared with the CMS + Vehicle group (*p* < 0.01; Fig. 3b).

**Fig. 4:**
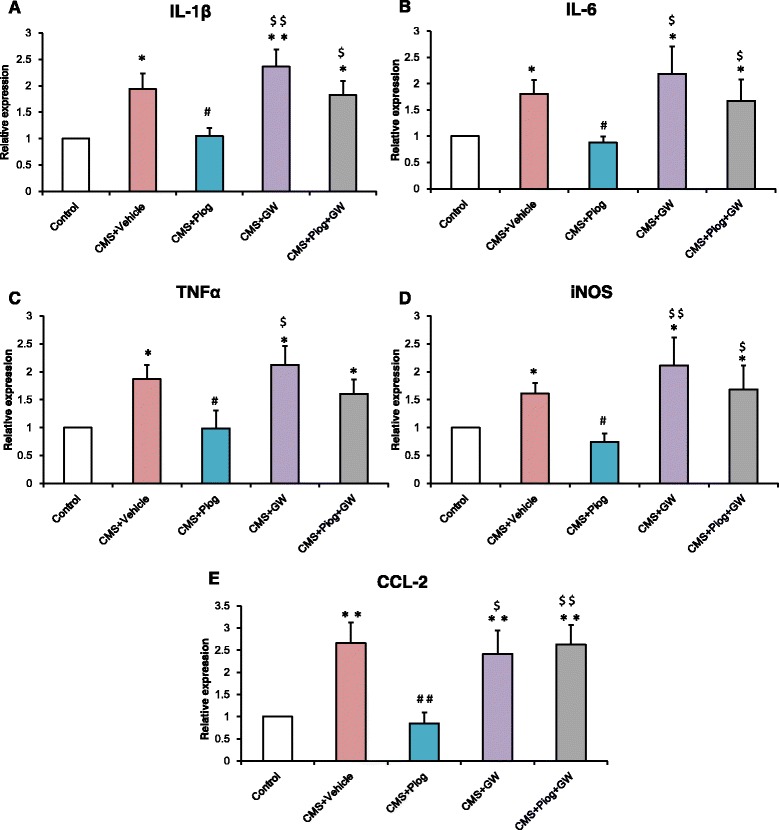
Effect of pioglitazone treatment on microglia-inhibited M1 activation in the DG of the hippocampus. The expression of M1 markers IL-1β (**a**), IL-6 (**b**), TNFα (**c**), iNOS (**d**), and CCL2 (**e**) increased; pioglitazone reduced this increase and GW9662 aggravated this increase in the CMS mice. *n =* 5. ^*^ *p* < 0.05, ^**^ *p* < 0.01 vs. Control; ^#^ *p* < 0.05, ^##^ *p* < 0.01 vs. CMS + Vehicle; ^$^ *p* < 0.05, ^$$^ *p* < 0.01 vs. CMS + Piog. Data are expressed as means ± SEM

**Fig. 5:**
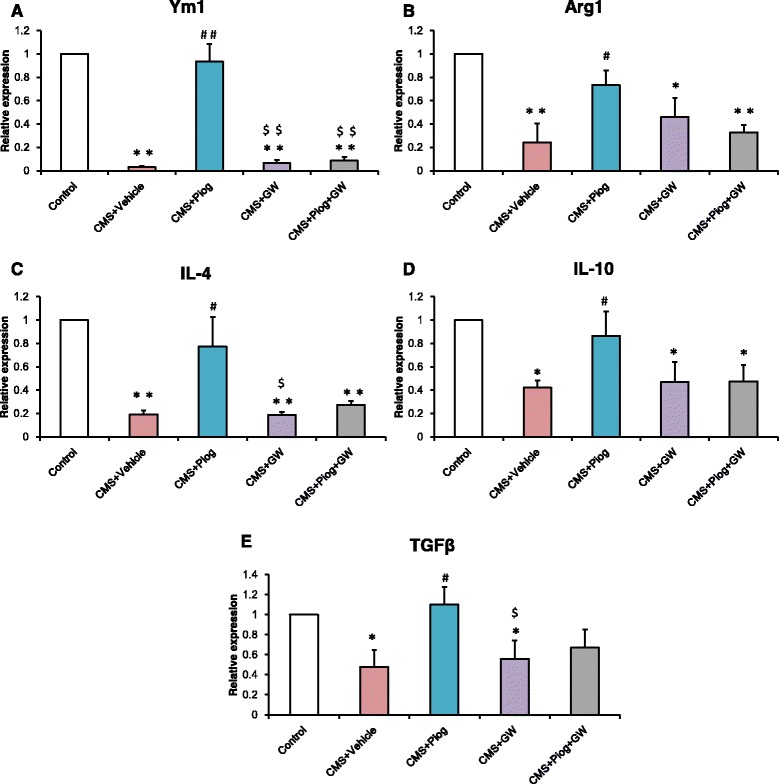
Effect of pioglitazone treatment on the microglial shift toward an M2 phenotype. The mRNA expression of M2: Ym1 (**a**), Arg1 (**b**), IL-4 (**c**), IL-10 (**d**), and TGFβ (**e**) reduced with a 6-week duration of CMS stress. The decreases were attenuated by pioglitazone administration. GW9662 still reduced the expression of these markers. Pioglitazone and GW9662 treated together had no effect on the M2 level of CMS mice. *n =* 5. ^*^ *p* < 0.05, ^**^ *p* < 0.01 vs. Control; ^#^ *p* < 0.05, ^##^ *p* < 0.01 vs. CMS + Vehicle; ^$^ *p* < 0.05, ^$$^ *p* < 0.01 vs. CMS + Piog. Data are expressed as means ± SEM

We next studied the activated phenotype of the microglia in the hippocampus. The expression of M1 markers, IL-1β, IL-6, TNFα, iNOS, and CCL2, increased in the CMS + Vehicle mice. After treatment with pioglitazone, the expression of M1 markers decreased. Administration of pioglitazone and GW9662 did not change the activation status of the microglia in the CMS animals (Fig. 4a: *p* = 0.018; Fig. 4b: *p* = 0.051; Fig. 4c: *p* = 0.045; Fig. 4d: *p* = 0.008; Fig. 4e: *p* = 0.032). In the M2 status, the messenger RNA (mRNA) expression (Ym1, Arg1, IL-4, IL-10, and TGFβ) was lower following the 6-week CMS procedure. The decreases were attenuated by administration with pioglitazone (Fig. 5a: *p* = 0.001; Fig. 5b: *p* = 0.004; Fig. 5c: *p* = 0.001; Fig. 5d: *p* = 0.037; Fig. 5e: *p* = 0.046).

### The effects of pioglitazone on LPS-stimulated N9 microglial phenotypes in vitro

**Fig. 6:**
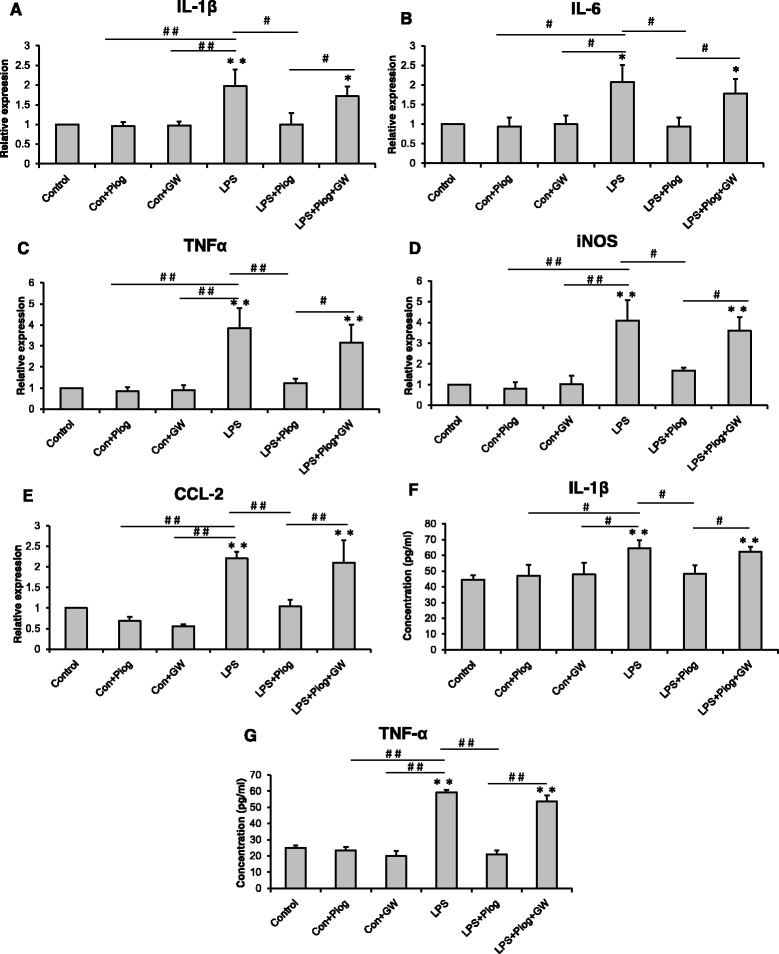
LPS-induced M1 polarization was alleviated by the PPARγ agonist pioglitazone in N9 microglial cells. LPS increased the level of IL-1β (**a**), IL-6 (**b**), TNFα (**c**), iNOS (**d**), and CCL2 (**e**), but this was reversed by pioglitazone. The PPARγ antagonist GW9662 inhibited the effect of pioglitazone on the PPARγ of microglia. The protein expression of IL-1β (**f**) and TNFα (**g**) was detected by ELISA. ^*^ *p* < 0.05, ^**^ *p* < 0.01 vs. Control; ^#^ *p* < 0.05, ^##^ *p* < 0.01. Data are expressed as means ± SEM

**Fig. 7:**
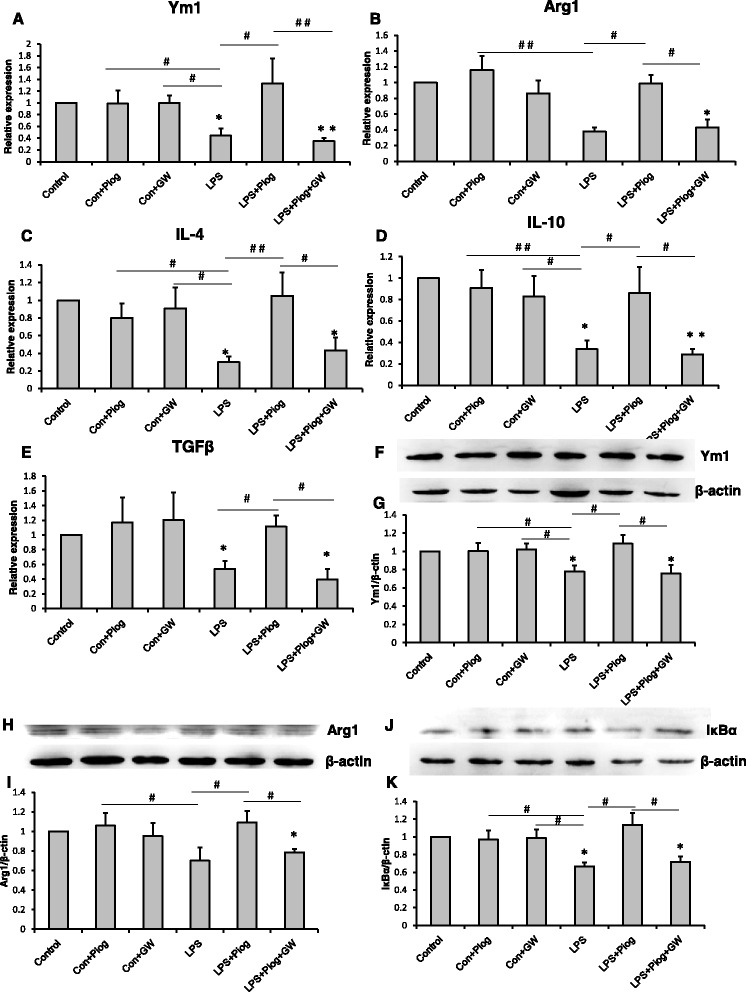
The M2 molecules: Ym1 (**a**), Arg1 (**b**), IL-4 (**c**), IL-10 (**d**), and TGFβ (**e**) decreased with LPS treatment. Pioglitazone increased the expression of the M2 markers. The impact on the LPS+Piog+GW group was similar to that of the LPS microglial cells. The level of Ym1 (**f** & **g**) and Arg1 (**h** & **i**) was also confirmed by western blot. The protein expression of IκBα decreased in LPS-stimulated cells; pioglitazone significantly ameliorated the expression. GW9662 suppressed the rise after pioglitazone-treated LPS in microglia (**j** & **k**).^*^ *p* < 0.05, ^**^ *p* < 0.01 vs. Control; ^#^ *p* < 0.05, ^##^ *p* < 0.01. Data are expressed as means ± SEM

In order to confirm the effect of pioglitazone on the microglial phenotypes, we detected the microglial activation status by using LPS simulate an organism’s inflammatory environment in an N9 microglial line. As shown in Fig. 6, the expression of the M1 phenotype (IL-1β, IL-6, TNFα, iNOS, and CCL2) increased significantly after the LPS treatment compared with the control, Con + Piog, and Con + GW microglial cells. Pioglitazone markedly blocked the upregulation of these markers in the LPS groups. Co-treatment with pioglitazone, GW9662, and LPS showed no difference in the LPS microglia (Fig. 6a: *p* = 0.025; Fig. 6b: *p* = 0.023; Fig. 6c: *p* = 0.001; Fig. 6d: *p* = 0.001; Fig. 6e: *p* = 0.036). In contrast, the expression of the M2 markers, Ym1, Arg1, IL-4, IL-10, and TGFβ, decreased after LPS stimulation, but these expressions improved after treatment with pioglitazone. The microglial activated phenotypes of the LPS + Piog + GW group were similar to the LPS microglia (Fig. 7a: *p* = 0.010; Fig. 7b: *p* = 0.012; Fig. 7c: *p* = 0.025; Fig. 7d: *p* = 0.040; Fig. 7e: *p* = 0.032). Consistent with these mRNA expressions, protein expression of the M1 pro-inflammatory cytokines (IL-1β and TNFα) was also upregulated by LPS, and these effects were significantly inhibited by pioglitazone. There were no differences between the LPS and the LPS + Piog + GW cells (Fig. 6f: *p* < 0.001; Fig. 6g: *p* < 0.001). The protein levels of the M2 anti-inflammatory mediators (Ym1 and Arg1) were also reduced with LPS treatment, and these decreases were attenuated by treatment with pioglitazone. There was no difference between the LPS and the LPS + Piog + GW microglia in their protein expression of either Ym1 or Arg1 (Fig. 7g: *p* = 0.041; Fig. 7i: *p* = 0.026). The results for the M1 and M2 markers in the N9 microglia cells in vitro were consistent with the consequences in the mice in vivo.

Next, we investigated whether pioglitazone exerted its anti-inflammatory effects through regulating NF-kB activity. Following the LPS treatment, the level of IkBα in the N9 microglia dropped compared with all the control groups. Pioglitazone significantly increased the levels of IkBα in the LPS-treated N9 cells, and GW9662 reversed this effect. Pioglitazone markedly upregulated the protein levels of IkBα, which repressed the inhibition of NF-kB activity (Fig. 7k: *p* = 0.026).

## Discussion

In this study, we investigated the antidepressant effect of pioglitazone and explored its underlying anti-inflammatory responses. First, pioglitazone ameliorated depression-like behaviors in CMS-treated mice, but this amelioration was diminished by GW9662. Second, pioglitazone exerted antidepressant effects through the anti-inflammatory activity of the PPARγ pathway. Third, pioglitazone improved CMS-induced depression-like behavior by regulating which of the microglial phenotypes (M1 or M2) was activated.

MDD is a common and sometimes fatal disorder that has increasingly become a public health concern. Accumulating evidence suggests that chronic low-grade inflammation plays an important role in the pathology of depression. Over the past two decades, great effort has been made to identify novel targets for antidepressant therapies. It has been proposed that pioglitazone, working as an anti-inflammatory agent, could produce antidepressant responses in patients with concomitant metabolic syndrome and diabetes [36, 37]. In our study, we assessed the antidepressant efficacy of pioglitazone on a CMS model, which is commonly used to induce and measure depression (Fig. 1). Because pioglitazone can cross the blood brain barrier, intragastric injection was selected for the CMS mice. Pioglitazone increased the SP in CMS-induced depressant model, which was blocked by the PPAR-γ antagonist GW9662. Pioglitazone as well decreased the time of immobility in FST and TST in the CMS-treated mice, indicating that pioglitazone has a therapeutic action in CMS-induced depression-like behaviors. CMS induced a decrease in locomotor activity indicated a depressive state of animals because of a loss of interest for the movement, or for exploring the environment. Pioglitazone increased spontaneous motor activity in the CMS-exposed mice, without effect on locomotor activity in normal mice [38]. Gavage administration of pioglitazone was effective at the 2.5 and 5.0 mg/kg doses for ameliorating the symptoms of depression, but the 2.5 mg/kg dosage was more effective. Dosages of 10 and 20 mg/kg were not efficacious in the CMS mice. As a result, the 2.5 mg/kg dose was chosen to treat the CMS mice in the subsequent experiments.

PPARγ is a ligand-dependent transcription factor belonging to the nuclear hormone receptor superfamily implicated in adipocyte differentiation, insulin sensitivity, and inflammatory processes [39, 40]. PPARγ is constitutively expressed in macrophages and CNS-resident microglia, acting as a key regulator of microglial activation [41]. Activation of PPARγ signaling has a protective role by reducing neuroinflammation [42], a finding which may present a novel therapeutic approach for diseases, including Alzheimer’s disease [20, 43], Parkinson’s disease [24, 44], stroke [45], schizophrenia, and autism [22]. In light of the anti-inflammatory and neuroprotective activities of the PPARγ-dependent signaling pathway, we investigated the antidepressant effects of pioglitazone in a CMS mice model of depression. GW9662, a selective antagonist for PPARγ, was administered in this investigation in order to explore the possible role of PPARγ activation on the antidepressant activities of pioglitazone (Fig. 2). After treatment with GW9662, the BW loss was greater than that in the CMS-treated mice. GW9662 significantly inhibited the pioglitazone-induced reduction in the duration of immobility in the FST and TST. GW9662 also counteracted the effect of pioglitazone on PPARγ activation. These results suggest that pioglitazone exerts antidepressant-like effects at least in part due to the activation of the PPARγ pathway.

Microglia play a crucial role in inflammation modulation in the CNS, and microglial dysfunction is believed to result in CNS immune disorders [46, 47]. The role of microglia has long been considered in some neurodegenerative diseases [48, 49]. Some interesting evidence about this role in depression has also began to emerge in recent years, e.g., microglial activation and the expression of inflammatory mediators have been reported in an olfactory bulbectomised (OB) rat model of depression [50]. According to in vitro research, microglia and their polarization status play an essential role in depression initiation [51]. These data indicated that the immune system was activated in the stressed depression models. This may have been due to the activation of M1 microglia [35]. Here, we showed that the microglial cells had large somas, short thick processes, and the amoeboid morphology typical of activated microglia after CMS initiation (Fig. 3a). This morphology has been explained in our previous experiments [12, 35]. Microglial activated phenotypes were determined by their polarized markers. We confirmed that the M1 markers (IL-1β, IL-6, TNFα, iNOS, and CCL2) were induced and that the M2 molecules (Ym1, Arg1, IL-4, IL-10, and TGFβ) were impaired in depression. Pioglitazone restored the balance of the M1 and M2 microglia in the hippocampus of the CMS mice (Figs. 4 and 5). The hippocampus has been implicated in the inhibition of stress responses [52] and in the regulation of affective states and emotional behavior [53]. Prior studies have shown that stress primed neuroinflammatory processes characterized by microglial activation [14, 15]. Microglial activation plays a vital role in the pathogenesis of many neurodegenerative diseases. Experiments in animal models have provided more direct evidence for a role of activated microglia in depression [50, 54]. In our experiment, we found that pioglitazone can control microglia activation and neuroinflammation in CMS-induced depression. These data indicated that microglia-modulating agents had therapeutic benefits for MDD, thereby defining a new biological activity for pioglitazone by showing that pioglitazone acts in modulating microglial phenotypes in a CMS-induced rodent model of depression.

Inflammatory cytokines play a significant role in depression. In our previous study, an imbalance between the pro- and anti-inflammatory cytokines may be one of the pathogeneses of depression [6]. In this current research, a higher expression of IL-1β, IL-6, and TNFα, and a lower expression of IL-4, IL-10, and TGF-β were observed in the hippocampus of CMS-induced mice (Figs. 4 and 5). A relatively large body of evidence has suggested that the PPARγ agonist pioglitazone can regulate the inflammatory response and oxidative stress [55, 56]. After pioglitazone treatment of the CMS mice, the expression of pro-inflammatory molecules was reduced and the levels of anti-inflammatory cytokines were increased. These results were consistent with recent findings that PPARγ agonists can transition M1 into M2 phenotype as well as enhance the production of anti-inflammatory cytokines such as IL-10 and TGFβ, both of which are pro-neurogenic [57, 58]. In order to ensure the specific response of activated microglia in stress-induced depression, experiments using LPS treatment of N9 microglial cells were performed. LPS can cause behavioral changes that indicate depression [59]. LPS stimulation can break the balance of microglial activation (M1 vs. M2), but the resulting imbalance in the levels of mRNA and protein can be ameliorated by pioglitazone administration (Fig. 6 and Fig. 7a–i). The results of an in vitro study were consistent with the mice test. Thus, the M1-M2 imbalance should be a focus in studying animal models of depression [50]. NF-kB pathway activation plays an important role in pro-inflammatory gene expression after stimulation [60, 61]. NF-kB normally exists in the cytoplasm, binding to its inhibitory proteins (IkB) and remaining inactive. Dissociation of IkB induces activation of NF-kB and facilitates the transcription of inflammatory genes [62]. In our experiment, the protein expression of IkB was reduced after an LPS stimulation of N9 microglial cells. Pioglitazone upregulated the IkBα expression in the LPS-treated N9 cells and GW9662 reversed the inhibitory effects of pioglitazone on NF-kB activity (Fig. 7k). These results indicated that the role of pioglitazone in preventing the NF-kB activation was partially linked to the upregulation of IkB expression. The present findings suggest that the anti-inflammatory effects of pioglitazone are associated with a PPARγ-mediated suppression of the NF-kB signaling pathway with consequential inflammatory cytokine expression in microglial cells.

## Conclusions

In summary, the present study demonstrated for the first time that the chronic administration of pioglitazone induced the neuroprotective phenotype of microglia in parallel with the amelioration of depression-like behaviors in CMS-treated C57BL/6 mice. Pioglitazone, a microglia-modulating drug which regulates anti-inflammatory activity, may partially account for the observed antidepressant response. This finding suggests that targeting microglia could pave the way for new depression treatments.

## Additional files

Additional file 1: BW and SP ratio in different weeks of experiment 1. (PDF 77 kb)
Additional file 2: BW and SP ratio in different weeks of experiment 2. (PDF 77 kb)

## Abbreviations

ANOVA: Analysis of variance
Arg1: Arignase1
BW: Body weight
CMS: Chronic mild stress
CNS: Central nervous system
FST: Forced swimming test
GW: GW9662
Iba1: Ionized calcium-binding adaptor protein-1
IkB: Inhibitor of NF-kB
LPS: Lipopolysaccharide
M1: Classical activation
M2: Alternative activation
MAOIs: Monoamine oxidase inhibitors
MDD: Major depressive disorder
NF-kB: Nuclear factor kB
OB: Olfactory bulbectomised
Piog: Pioglitazone
PBS: Phosphate-buffered saline
PPARγ: Peroxisome proliferator-activated receptor γ
RT-PCR: Real time-PCR
SP: Sucrose preference
SSRIs: Selective serotonin reuptake inhibitors
TNFα: Tumor necrosis factor-α
TST: Tail suspension test
