# Supplementary material for: The antidepressant-like effects of pioglitazone in a chronic mild stress mouse model are associated with PPARγ-mediated alteration of microglial activation phenotypes
Source: J Neuroinflammation. 2016 Oct 4;13:259. doi: 10.1186/s12974-016-0728-y (PMC5051050; doi:10.1186/s12974-016-0728-y)
Supplement: Additional file 1: — BW and SP ratio in different weeks of experiment 1. (PDF 77 kb) [file 12974_2016_728_MOESM1_ESM.pdf]

BW and SP ratio in different weeks of experiment 1.

	Week 0		Week 1		Week 2		Week 3		Week 4		Week 5		Week 6	
**Group**	BW (g)	SP (%)	BW (g)	SP (%)	BW (g)	SP (%)	BW (g)	SP (%)	BW (g)	SP (%)	BW (g)	SP (%)	BW (g)	SP (%)
Control	20.59±0.88	84.3±2.9	21.63±1.38	68.3±8.5	22.06±1.21	72.9±2.6	22.60±1.36	77.9±4.8	23.15±0.73	84.1±9.2	23.36±1.01	86.1±4.7	25.22±0.84	88.8±6.2
CMS+Vehicle	20.60±0.98	84.8±3.5	20.35±1.43	69.6±7.5	20.38±0.76	73.0±5.2	20.55±0.80	75.7±5.4	21.03±1.09	76.4±2.3	21.25±1.23	79.5±6.3	21.48±0.90	79.5±5.8
CMS+2.5mg/kg	20.56±0.74	84.9±6.7	21.11±0.86	75.5±7.6	21.73±1.61	81.7±4.8	21.93±0.65	77.9±4.5	22.18±1.12	78.6±4.7	22.61±1.33	83.8±9.8	24.10±0.79	87.2±8.8
CMS+5.0mg/kg	20.59±0.84	82.7±7.2	21.10±0.94	68.5±5.8	21.63±1.44	79.4±6.4	21.66±1.41	78.3±7.4	21.99±0.73	84.1±10.2	22.33±1.39	82.7±8.4	23.62±0.57	87.2±6.3
CMS+10mg/kg	20.63±1.21	82.7±6.1	21.05±0.88	67.5±4.7	21.48±0.72	80.1±7.4	21.29±1.21	79.2±9.5	21.74±0.85	84.8±9.5	21.87±1.30	80.1±7.5	23.40±0.88	81.8±8.5
CMS+20mg/kg	20.63±0.91	81.1±8.5	20.70±1.01	68.7±6.8	21.28±0.96	81.5±5.3	20.55±1.03	73.4±6.1	21.54±1.00	80.4±8.6	21.94±1.41	73.9±9.7	23.26±0.58	77.3±10.4
p values	p>0.05	p>0.05	p>0.05	p>0.05	p>0.05	p>0.05	p>0.05	p>0.05	p>0.05	p>0.05	p>0.05	p>0.05	p<0.05	p<0.05
