# Supplementary material for: The antidepressant-like effects of pioglitazone in a chronic mild stress mouse model are associated with PPARγ-mediated alteration of microglial activation phenotypes
Source: J Neuroinflammation. 2016 Oct 4;13:259. doi: 10.1186/s12974-016-0728-y (PMC5051050; doi:10.1186/s12974-016-0728-y)
Supplement: Additional file 2: — BW and SP ratio in different weeks of experiment 2. (PDF 77 kb) [file 12974_2016_728_MOESM2_ESM.pdf]

BW and SP ratio in different weeks of experiment 2.

	**Week 0**		**Week 1**		**Week 2**		**Week 3**		**Week 4**		**Week 5**		**Week 6**	
**Group**	BW (g)	SP (%)	BW (g)	SP (%)	BW (g)	SP (%)	BW (g)	SP (%)	BW (g)	SP (%)	BW (g)	SP (%)	BW (g)	SP (%)
Control	20.57±1.12	80.2±8.2	21.47±1.08	68.3±3.9	22.55±0.70	72.9±4.2	23.16±0.61	77.9±3.9	23.58±0.95	84.1±4.2	24.03±0.82	86.1±3.6	25.22±0.78	80.7±4.7
CMS+Vehicle	20.58±1.05	80.0±7.8	21.27±0.95	69.6±2.9	21.96±0.45	73.0±6.2	22.23±0.68	75.7±5.4	22.87±0.92	76.4±9.5	23.08±0.91	79.5±4.7	23.70±0.34	62.5±9.5
CMS+Piog	20.52±0.95	79.4±9.5	21.21±0.68	75.5±4.5	21.76±0.24	81.7±2.2	21.98±0.53	77.9±2.5	22.67±0.74	78.6±4.5	23.52±0.48	83.8±4.4	25.08±0.71	79.0±10.2
CMS+GW	20.58±0.79	81.2±5.9	21.27±0.80	68.5±8.6	21.72±0.62	79.4±8.5	18.63±0.49	78.3±4.2	18.85±1.21	84.1±4.6	20.42±0.73	82.7±3.8	21.17±0.56	70.5±6.8
CMS+ Piog+GW	20.62±0.53	79.4±3.8	21.12±0.72	67.5±11.2	21.41±0.89	80.1±8.1	19.66±0.69	79.2±6.0	20.73±0.43	84.8±3.2	21.11±0.66	80.1±4.7	21.93±0.60	72.1±12.1
p values	p>0.05	p>0.05	p>0.05	p>0.05	p>0.05	p>0.05	p>0.05	p>0.05	p>0.05	p>0.05	p>0.05	p>0.05	p<0.05	p<0.05
